# Clinicopathological features and prognosis of gastric adenosquamous carcinoma

**DOI:** 10.1038/s41598-017-04563-2

**Published:** 2017-07-04

**Authors:** Fan Feng, Gaozan Zheng, Jingpeng Qi, Guanghui Xu, Fei Wang, Qiao Wang, Man Guo, Xiao Lian, Hongwei Zhang

**Affiliations:** 1 Department of Digestive Surgery, Xijing Hospital, Fourth Military Medical University, 127 West Changle Road, 710032 Xi’an, Shaanxi China; 2 Department of Oncology, First Affiliated Hospital, Xi’an Medical University, 48 West Fenggao Road, 710077 Xi’an, Shaanxi China

## Abstract

Data about primary gastric adenosquamous carcinoma (ASC) was limited due to rare incidence. Thus, the present study aims to investigate clinicopathological features and prognosis of gastric ASC. Cases of gastric ASC were obtained from our center and from case reports and series extracted from Medline. Clinicopathological features and prognosis of gastric ASC were analyzed and compared with gastric adenocarcinoma (AC) in our center. The commonest location was lower third (45.0%), followed by upper (26.2%) and middle third (24.2%). The median tumor size was 6 cm (0.8–17). The commonest differentiation status was well for both AC (44.4%) and SCC components (40.9%). Half of tumors (52.7%) were stage T4 and most patients (86.2%) suffered from lymph node metastasis (LNM). Tumor depth and TNM stage were risk factors for overall survival (OS) (both P < 0.05). The distribution of age, tumor size, tumor location, tumor depth, LNM and TNM stage were significantly different between gastric ASC and AC (all P < 0.05). The OS of gastric ASC was significantly worse than AC (P < 0.001), especially in stage III disease (P < 0.001). Gastric ASC differ significantly from AC with respect to clinicopathological features. The prognosis of gastric ASC was worse than AC.

## Introduction

Gastric adenocarcinoma (AC) is the most common type of primary gastric cancer, whereas gastric primary adenosquamous carcinoma (ASC) is extremely rare. It accounted for less than 1% of all gastric cancers^1^. Gastric ASC is characterized by coexisting of two components (AC and ASC) within the same tumor^2^. Due to the rare incidence, gastric ASC was described in case reports and case series with small number of patients, study on gastric ASC with large series cases was lacking. Up to date, a variety of issues about gastric ASC remains unclear, including histogenesis, clinicopathological characteristics, optimal treatment strategies, and prognosis, etc. Thus, the present study aims to investigate the clinicopathological features and prognosis of gastric ASC based on a large series of cases.

## Results

### Clinicopathological characteristics of gastric ASC

The clinicopathological characteristics were summarized in Table 1. There were 121 male (73.3%) and 44 female (26.7%) patients. The median age was 63 years (range 26–88 years). Thirty-two patients (25.4%) accompanied with distant metastasis at the time of diagnosis. The commonest location was lower third (45.0%), followed by upper (26.2%) and middle third (24.2%). The median tumor size was 6 cm (range 0.8–17 cm). The commonest differentiation status for AC components was well differentiation (44.4%), followed by poorly (38.9%) and moderately differentiation (16.7%). The commonest differentiation status for SCC was well differentiation (40.9%), followed by moderately (34.1%) and poorly differentiation (25.0%). One hundred and twenty-three patients (78.9%) received complete resection, 25 patients (16.0%) received palliative resection, and 8 patients (5.1%) did not receive surgery. The distribution of T stage was 3.0% for T1, 16.8% for T2, 27.5% for T3 and 52.7% for T4. Most of the patients (86.2%) suffered from LNM. With respect to the components in metastatic LNs, AC was found in 58.7% of cases, SCC was found in 19.6% of cases, and both AC and SCC was found in 21.7% of cases.

**Table 1:** Clinicopathological features of gastric primary ASC.

Characteristics	Gastric ASC (n = 167)	Percentage
Age	∑ = 154	
≤60	65	42.2%
>60	89	57.8%
Gender	∑ = 165	
Male	121	73.3%
Female	44	26.7%
Distant metastasis	∑ = 126	
Yes	32	25.4%
No	94	74.6%
Tumor location	∑ = 149	
Upper third	39	26.2%
Middle third	36	24.2%
Lower third	67	45.0%
Two thirds or more	7	4.6%
Tumor size (cm)	∑ = 140	
≤5	58	41.4%
>5	82	58.6%
Differentiation status	AC ∑ = 18	SCC ∑ = 44
Well differentiated	8	18
Moderately differentiated	3	15
Poorly differentiated	7	11
Surgical resection	∑ = 156	
Complete resection	123	78.9%
Incomplete resection	25	16.0%
No surgery	8	5.1%
Tumor depth	∑ = 131	
T1	4	3.0%
T2	22	16.8%
T3	36	27.5%
T4	69	52.7%
Lymph node metastasis	∑ = 109	
N0	15	13.8%
N1	36	33.0%
N2	25	22.9%
N3	33	30.3%
Metastatic components in lymph node	∑ = 46	
A	27	58.7%
S	9	19.6%
A & S	10	21.7%
Adjuvant therapy	∑ = 131	
Yes	42	32.1%
No	89	67.9%

### Prognosis of gastric ASC

**Figure 1:**
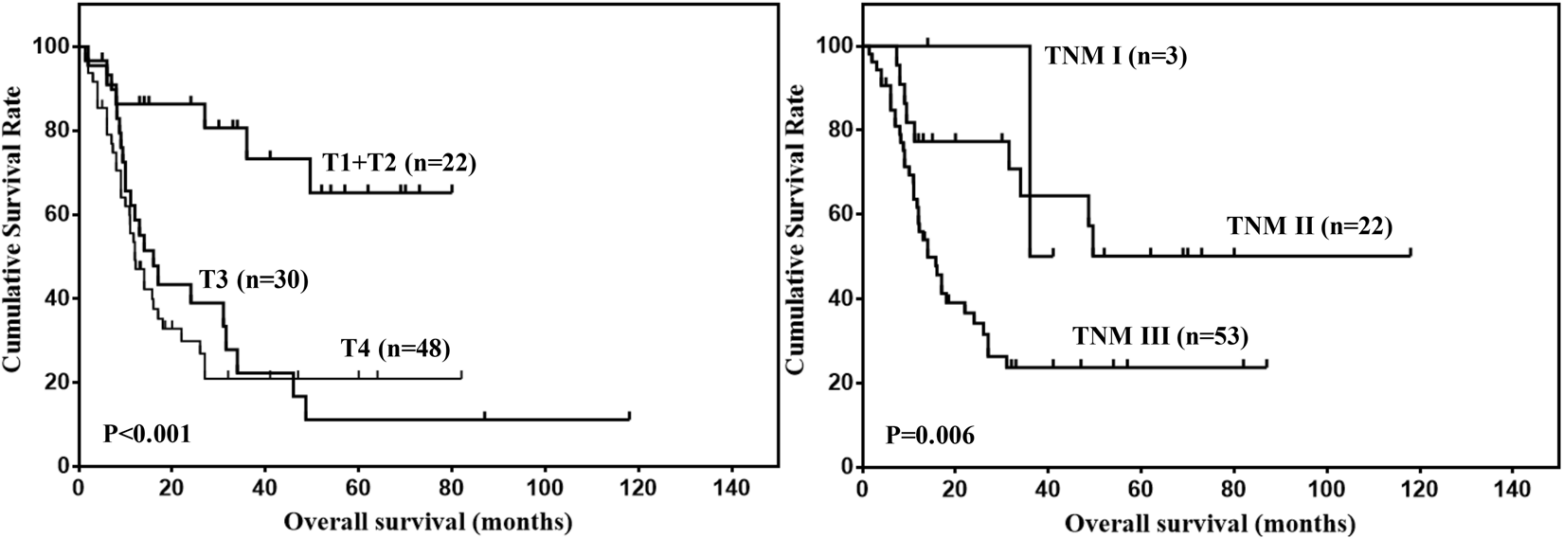
OS of gastric ASC stratified by tumor depth and TNM stage.

One hundred and nine patients with R0 resection and follow up data were selected for survival analysis. The median follow-up time was 33 months (range 5–118 months). The 1, 3 and 5-year OS was 58.1%, 32.4% and 26.4%, respectively. Prognostic predictors for patients were analyzed by univariate analysis (Table 2). The results showed that only tumor depth (P < 0.001) and TNM stage (P = 0.006) were prognostic risk factors. The OS stratified by tumor depth and TNM stage were shown in Fig. 1.

**Table 2:** Risk factors for OS of gastric ASC patients according to univariate analysis (n = 109).

Prognostic factors	β	Hazard ratio (95% CI)	P value
Age	−0.103	0.903 (0.546–1.492)	0.689
Gender	−0.473	0.623 (0.331–1.173)	0.143
Tumor location	−0.091	0.913 (0.689–1.208)	0.523
Tumor size	0.372	1.451 (0.879–2.396)	0.146
Differentiation status	−0.657	0.519 (0.172–1.559)	0.242
Tumor depth	0.490	1.633 (1.264–2.109)	<0.001
Lymph node metastasis	0.010	1.010 (0.488–2.088)	0.979
TNM stage	0.907	2.477 (1.301–4.718)	0.006

### Comparison of clinicopathological characteristics and prognosis between gastric ASC and AC

**Figure 2:**
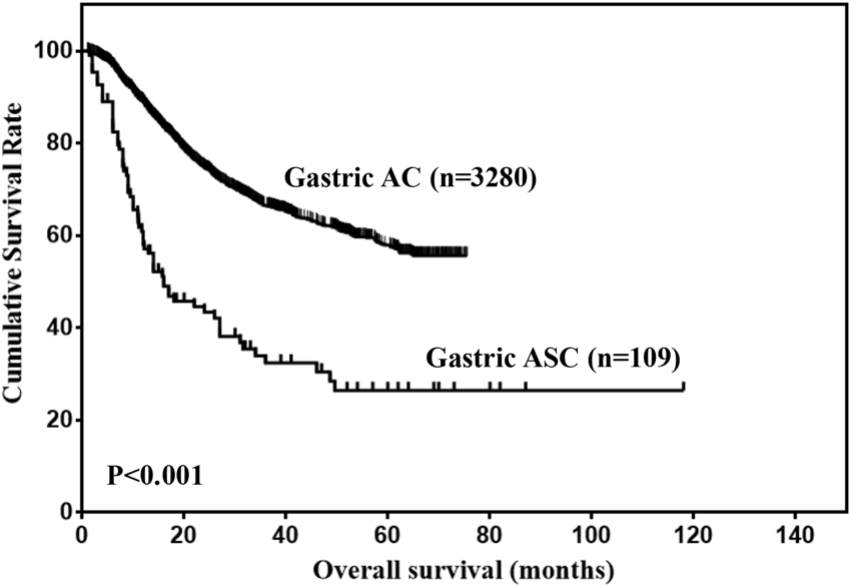
Comparison of OS between gastric ASC and AC for the entire cohort.

**Figure 3:**
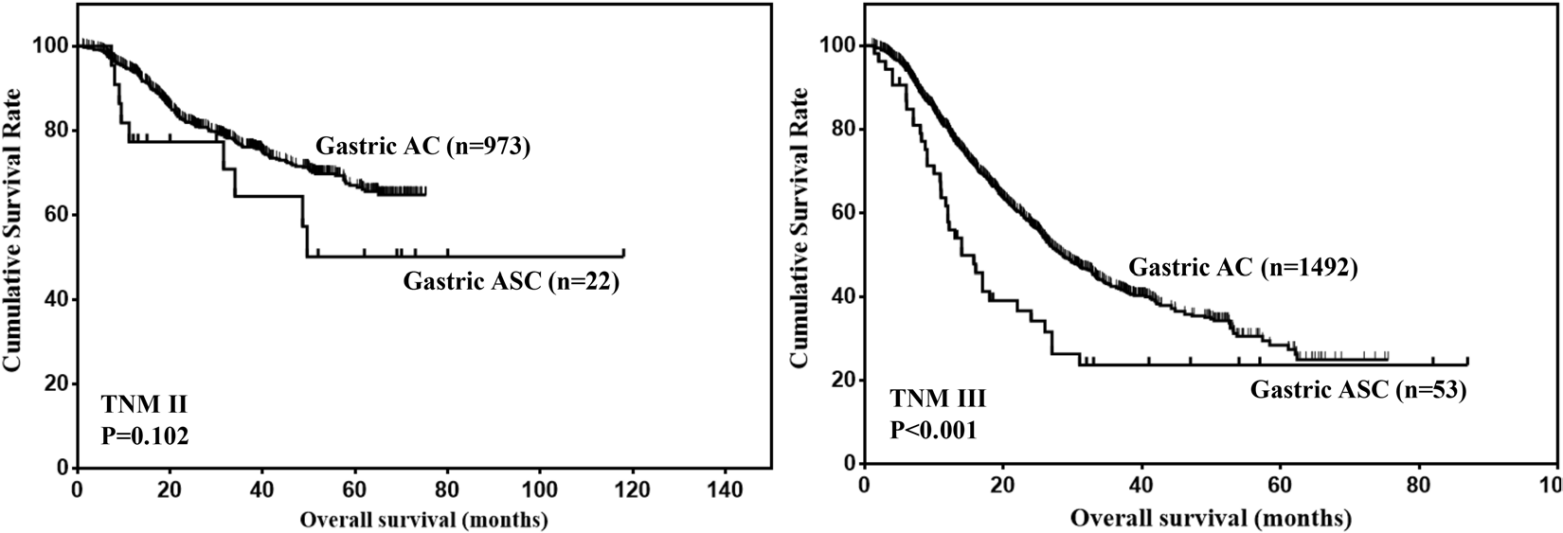
Comparison of OS between gastric ASC and AC in stage II/III patients.

The clinicopathological characteristics of 109 gastric ASC patients were compared with 3280 gastric AC patients in our center (Table 3). The results showed that the distribution of age, tumor size, tumor location, tumor depth, LNM and TNM stage were significantly different between gastric ASC and AC (all P < 0.05). Then, the prognosis of gastric ASC and AC were compared. The OS of gastric ASC was significantly worse than that of gastric AC (Fig. 2, Table 4, P < 0.001). Further, the OS of gastric ASC and AC with stage II/III disease were compared. The results showed that the prognosis of stage II gastric ASC was comparable to that of stage II gastric AC (Fig. 3, P = 0.102), and the prognosis of stage III gastric ASC was worse than that of stage III gastric AC (Fig. 3, P < 0.001).

**Table 3:** Comparison of selected clinicopathological parameters between gastric AC and ASC patients underwent R0 resection.

Characteristics	AC(n = 3280)	ASC(n = 109)	P value
Age			
≤60	1945	39	<0.001
>60	1335	62	
Gender			
Male	2546	83	0.297
Female	734	30	
Tumor location			
Upper third	1012	32	0.036
Middle third	541	28	
Lower third	1459	44	
Two thirds or more	268	4	
Tumor size (cm)			
≤5	2266	41	<0.001
>5	1014	61	
Tumor depth			
T1	612	2	<0.001
T2	515	20	
T3	1211	30	
T4	942	48	
Lymph node metastasis			
N0	1178	13	0.006
N1	639	21	
N2	560	17	
N3	903	27	
TNM stage			
I	815	3	<0.001
II	973	22	
III	1492	53

**Table 4:** Comparative overall survival analysis of gastric AC and ASC using univariate and multivariate analysis.

Overall survival	Gastric AC	Gastric ASC	Univariate analysis			Multivariate analysis		
	n = 3280	n = 109	β	HR (95% CI)	P	β	HR (95% CI)	P
			−1.094	0.335(0.263–0.427)	<0.001	−0.539	0.583(0.425–0.801)	0.001
1-year	89.0%	58.1%						
3-year	66.5%	32.4%						
5-year	57.9%	26.4%

## Discussion

Gastric ASC is an extremely rare entity and account for less than 1% of all gastric malignancies^1^. Thus, the clinicopathological features and prognosis of gastric ASC was unclear. In the present study, we found that gastric ASC differ significantly from gastric AC with respect to clinicopathological features, and the prognosis of gastric ASC was worse than that of gastric AC.

Up to date, there was only one study containing a relatively large number of gastric ASC patients^3^. The clinicopathological features of 120 cases of gastric ASC was reviewed in this study. In their series, the male to female ratio was 2.3:1, and the mean age was 58.4 years. The most common location was lower third, followed by middle third and upper third. However, the prognosis of gastric ASC was not analyzed. In our present study, the male to female ratio was 2.8:1, and the patient age ranged from 26 to 88 years (mean: 61.3 years, median: 63 years). The most common location was lower third, followed by upper third and middle third. The results in our study was inconsistent with the previous reports.

It is well accepted that the diagnosis of gastric ASC was confirmed by the coexisting of AC and SCC components, with SCC accounting for at least 25% of tumors^1^. However, Faria *et al*. proposed that tumors should be located outside the cardia, without esophageal invasion and without ASC in any other organs^4^. The histogenesis of gastric ASC was still under debate. Several hypotheses have been proposed:^5^ (1) squamous metaplastic transformation of AC, (2) oncogenic transformation of ectopic squamous epithelium, (3) oncogenic transformation of metaplastic squamous cells, (4) collision of concurrent AC and SCC, (5) differentiation of stem cells toward both glandular and squamous cells. The first hypothesis was supported by many researchers based on accumulating evidence. Firstly, most of the SCC components were located in deeper layer, in contrast to the AC components being located in the mucosal layer^6^. Secondly, an obvious transition area exists between AC and SCC components^1^. Thirdly, the positive expression of CEA was found in SCC components^1^. Fourthly, identical levels of p53 gene was found in both components^6, 7^.

Gastric ASC was an extremely aggressive tumor. Most of them are found in an advanced stage at the time of diagnosis^4, 8, 9^. In our present study, 25.4% of patients accompanied by distant metastasis. Among them, the most common location for distant metastasis was liver, followed by peritoneal dissemination. Half of the tumors (52.7%) were stage T4 and the incidence of LNM was 86.2%. These findings were all consistent with previous reports^1, 2, 10^.

Both AC and SCC components have the potential for distant metastasis. Lee *et al*. reported that AC components were found in 10 of 14 cases, SCC component was found in 1 patient, and both components were found in 3 patients^7^. Chen *et al*. also analyzed the metastatic LNs and revealed that AC was the major component in 6 cases, and SCC was the major component in one case^10^. A study containing 12 cases of gastric ASC with LNM also found that 8 cases had AC components, 2 cases had SCC components and 2 cases had both components^1^. In our present study, for patients with LNM, AC was found in 58.7% of cases, SCC was found in 19.6% of cases, and both AC and SCC was found in 21.7% of cases. Thus, AC may be the predominant component for LNM. However, Mori *et al*. reported that both components existed in almost all the metastatic lesions in 9 patients at autopsy^11^. In all, the incidence of different components in the metastatic LNs needs further investigation based on larger sample size.

Radical resection remains the optimal treatment for local disease without distant metastasis. However, no standard adjuvant therapy strategies for gastric ASC has been established. Chemotherapy has been reported to be effective for gastric ASC^12^. However, there is no consensus on the optimal strategy of chemotherapy. Radiotherapy could also be used as one of the adjuvant treatment, as the SCC components in gastric ASC was sensitive to radiotherapy^1^.

The prognosis of gastric ASC was considered to be worse than typical gastric AC, although the biological behavior was mainly determined by the AC components^13^. Quan *et al*. reported that the median overall survival time was 12 months, and 87.5% of patients survived for less than 24 months after diagnosis^2^. Chen *et al*. reported that the median overall survival time was 22 months, and 3-year overall survival rate was 15.4%^1^. However, these data were based on small number of patients, and not all patients received radical resection. In our present study, the median overall survival time was 17 months for gastric ASC received R0 resection, and the 1, 3 and 5-year overall survival rate was 58.1%, 32.4% and 26.4%, respectively. Further, we compared the overall survival of gastric ASC with gastric AC patients. We found that the prognosis of gastric ASC was significantly poorer than that of gastric AC patients.

There are several limitations in our present study. First, the sample size was not large enough. Thus, the results of our present study should be explained cautiously. Second, the completeness of data is limited due to data acquisition. Third, data about differentiation status of both AC and SCC components in the primary tumor was limited. Thus, the association between the differentiation status of both components and the prognosis of patients could not be evaluated. Fourth, the association between clinicopathological features and components in the metastatic lymph nodes could not be analyzed due to the limited data. Fifth, data about the components in the recurrent and metastatic lesions was lacking. The influence of components on the prognosis of patients was unclear. Sixth, the constituent ratio of AC and SCC components was varied among primary tumors. The prognostic value of constituent ratio on the prognosis of gastric ASC was unclear. Seventh, the association between the components in the metastatic lymph nodes and components in the recurrent and distant metastatic lesions was unclear. The last, the disease free survival and disease specific survival could not be evaluated due to the data acquisition.

In conclusion, the majority of tumors were located in the lower third, well differentiation, stage T4 and stage N+. Tumor depth and TNM stage were risk factors for overall survival. Gastric ASC differ significantly from gastric AC with respect to clinicopathological features. The prognosis of gastric ASC was worse than gastric AC.

## Methods

**Figure 4:**
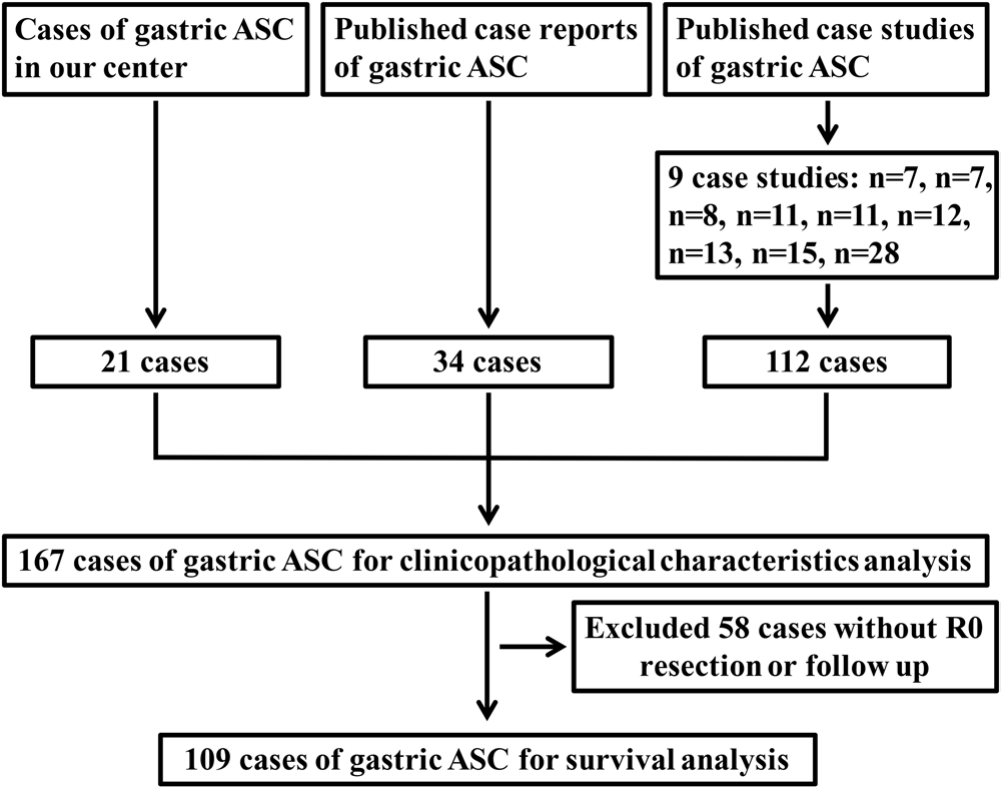
Flowchart of patient selection.

Gastric AC and ASC cases were from our center and literature. From September 2008 to March 2015, 21 cases of gastric ASC and 3280 cases of gastric AC received radical resection in our center. Literature search of Medline was performed for articles in English published from 1965 through 2015. Medline search resulted in 43 case reports and studies^1–43^ including 146 cases of gastric ASC. Finally, a total of 167 gastric ASC patients was identified (Fig. 4). This study was approved by the Ethics Committee of Xijing Hospital, all procedures performed in studies involving human participants were in accordance with the ethical standards of the institutional and/or national research committee and with the 1964 Helsinki declaration and its later amendments or comparable ethical standards. Informed consent was obtained from all individual participants included in the study.

Data including gender, age, distant metastasis, tumor location, tumor size, differentiation status, surgical intervention, tumor depth, LNM, adjuvant therapy and survival data were extracted from case reports and studies of recorded from our center. Completeness of data is limited due to the type of data acquisition. Patients in our center were followed up till November 2015 by enhanced chest and abdominal CT and gastroscopy every 3 months.

Data were processed using SPSS 22.0 for Windows (SPSS Inc., Chicago, IL, USA). Discrete variables were analyzed using Chi-square test or Fisher’s exact test. Significant prognostic predictors for patients identified by univariate analysis were further assessed by multivariate analysis using the Cox’s proportional hazards regression model. OS was shown by Kaplan-Meier method. The P value was considered to be statistically significant at 5% level.
